# Conflict between Farmers and Guanacos (*Lama guanicoe cacsilensis*): Field Surveys, Remote Sensing, and Interviews Provide Information for Conservation of a Critically Endangered Species in Southern Peru

**DOI:** 10.3390/ani14050658

**Published:** 2024-02-20

**Authors:** Hugo Castillo-Doloriert, Daniela Velasquez, Yumi Matsuno, Domingo Hoces, Jane C. Wheeler

**Affiliations:** 1 CONOPA—Instituto de Investigación y Desarrollo de Camélidos Sudamericanos, Lima 15823, Peru; danielavelasdvc@gmail.com (D.V.); ymatsuno.r@gmail.com (Y.M.); domingoh2647@yahoo.com (D.H.); jcwheeler@conopa.org (J.C.W.); 2 Facultad de Medicina Veterinaria, Universidad Nacional Mayor de San Marcos, Lima 15039, Peru; 3 IUCN SSC South American Camelid Specialist Group, 1196 Gland, Switzerland

**Keywords:** guanaco, farmers, conflict, Peru

## Abstract

**Simple Summary:**

The South American landscape has changed since the Spanish conquest, largely due to the introduction of foreign plants and animals, often at the expense of native species. In the south, Patagonia became a world-class sheep production center, and the native guanaco has almost disappeared. In Peru, guanacos are critically endangered, and an important local increase in their numbers is presently causing a conflict with local farmers in two southern communities. We studied guanaco movements in the area over a year using information from field surveys and remote sensing data from GPS-collared animals. We found that guanaco family groups did not invade agricultural fields, but free-ranging bands of young males and single adults did, perhaps pushed by the greater number of animals and decreased food resources. Information about the losses suffered by the farmers was obtained through interviews. The vast majority felt that guanacos are a problem to be resolved by building better fences, increasing security, or even illegal hunting. Although they are aware that guanacos are protected by law, 92% saw no present or future value in protecting the guanaco. These results show how much research and public education remain to be done to protect the Peruvian guanaco.

**Abstract:**

The Peruvian guanaco (*Lama guanicoe cacsilensis*) is classified as being “in critical danger of extinction” by the government. In this study, we evaluate how the conflict between farmers and guanacos in the Susapaya and Estique Districts, Tacna Department (Southern Peru) may represent a threat to their survival. To evaluate the situation, we 1. Conducted field surveys to monitor guanaco presence, 2. Used available remote sensing data to map guanaco movement, and 3. interviewed the impacted farmers concerning their losses. Remote sensing data showed that sedentary guanaco family groups located in prime steppe vegetation habitats never entered agricultural areas, while field surveys showed that bachelor bands and solitary individuals did, perhaps seeking forage due to growing population pressure. Interview data found that 90% of community farmers felt that guanacos were a problem best resolved by better fencing (45%), hunting (19%), or increased security (16%), and 92% saw no value in the conservation of the species. Hunting is illegal, given the critically endangered status of guanacos in Peru, so additional efforts are needed to both educate those who feel guanacos are a menace and involve them in efforts to preserve the species.

## 1. Introduction

Human–wildlife conflict is defined as negative interaction between human and wildlife populations when their differing priorities and/or behaviors exert pressure on the landscape [1]. Conflict emerges when the presence or behavior of a wild species represents a direct, recurring threat to real or perceived human needs or interests, resulting in negative impacts on both the human and wildlife populations [2]. It most commonly results from human activities such as land clearance, usually spreading from urban centers into rural areas and reducing wildlife habitat [3], but may also result from an increase in the size of wildlife populations [4].

DNA analysis has determined the existence of two subspecies of *guanicoe*, *Lama guanicoe guanicoe* Müller, 1776, in Argentina, Paraguay, Bolivia, and Chile, and *Lama guanicoe cacsilensis* Lönnberg, 1913, in Peru and northern Chile [5,6,7] and recorded two major genetic bottlenecks ~2000 years ago [8] and around the time of the Spanish invasion ~500 years ago [9]. Current guanaco distribution extends from northern Peru (*L. g. cacsilensis*), south along the Andes to Tierra del Fuego, and across the continent to the east (*L. g. guanicoe*), with body size increasing from adult animal withers heights from 100 to 120 cm; body length from the tip of the nose to the base of the tail from 90 to 191 cm; and live weight from 96 to 130 kg across the north/south gradient [10]. Guanacos have adapted to extreme environments from the hyper-arid Atacama Desert to the perpetually wet forests of Tierra del Fuego and are found in four of the ten major South American habitats: desert and xeric shrublands, montane and lowland grasslands, savannas and shrublands, and wet temperate forests [11]. They are not strict grazers, preferring browse when available, and are distributed at elevations from sea level to 4500 m in the high Andes [10,11].

The guanaco *Lama guanicoe* is classified as a species of least concern by the IUCN due to the large population (~2,000,000) of the subspecies *L. g. guanicoe* located principally in Patagonia [12]. Research on the subspecies *L. g. guanicoe* provides detailed information concerning the biology and genetics [5,6,7], spatial ecology [13,14,15,16], diet [17,18,19], and the conflict with intensive sheep rearing [20,21,22] in southern South America that have provided a sound scientific base for proposing a National Conservation Strategy in Argentina [23], and the development of a live shearing for fiber production program that is considered a viable conservation practice [24].

In contrast, the northern subspecies, *Lama guanicoe cacsilensis* is classified as critically endangered by the Peruvian government (DS No. 004-2014-MINAGRI), based on a total estimated population of between 3000 and 5000 animals [25]. The last national census was conducted in 1996 [26] and, in 2006, an international population and habitat viability assessment PHVA workshop held in Lima found that extinction could occur in 30 years if hunting was not curtailed [27]. Initial research has found high levels of genetic variability in some populations [6,8], but this does not ensure their survival, nor does it support captive rearing with shearing for fiber production as a conservation strategy until more information on the genetic situation of the drastically reduced populations is available [6].

Some Peruvian guanaco populations have recently undergone considerable growth. At the northernmost limit of their distribution in La Libertad Department, census data from the Calipuy National Reserve record an increase from 538 in 1996 to 1862 in 2023 [28], and at the southernmost limit, the number of unprotected, free-ranging guanacos in Tacna Department was 95 in 1996, 52 in 2009, and 406 in 2015 [26,29,30]. The majority of Tacna Department guanacos are located in Tarata Province, where a major increase has been recorded in the last seven years [30], leading to the conflict situation with local farmers described here.

Across their distribution, guanaco population ranges overlap with domestic livestock grazing lands, agricultural fields, and mining activities, creating areas of direct conflict with human activities [20,31,32]. Documentation of the human–guanaco conflict has focused on presumed competition between guanacos and sheep, a non-native introduced domestic livestock that dominates the steppe landscape across much of southern South America where guanaco numbers are greatest [20,31].

In Patagonia, since the massive introduction of sheep in the 1800s, drought, overgrazing, and poor management practices have all contributed to environmental degradation and ongoing desertification; yet, there is a generalized perception among ranchers that increased guanaco numbers contribute importantly to decreased forage resources [33,34]. In a recent study, Flores et al. [20] found that the negative perception of guanacos held by sheep herders of Isla Grande (Tierra del Fuego, Argentina) is based on their belief that increased guanaco presence has resulted in reduced pasture cover. Nonetheless, it was found that those who most strongly blamed guanacos also overestimated their population size. Further north, in central Chile, both climate change induced aridity and guanaco population growth are at the center of an increasing conflict, with herders seeking greater access to pastoral resources for their domestic livestock [3,32].

In 2019, Oliva et al. [21] concluded that at the end of the last century, after an extended period of overgrazing, the domestic livestock of Patagonia had approached carrying capacity, but the increase of guanaco numbers has subsequently pushed some areas above carrying capacity. This conclusion was rejected by Marino et al. [22] as lacking empirical support because grazing is not really at equilibrium, as sheep remain concentrated in overgrazed areas. Of particular importance in this article is the conclusion that guanacos utilize marginal areas that are rarely grazed by, or are inaccessible to, sheep, thereby creating and occupying a different niche than that occupied by the sheep, so that even if guanaco numbers were reduced, grassland degradation and production losses would continue because their main drivers, domestic overstocking and heterogeneous grazing, are still operating.

Although negative interactions between guanacos and agricultural, agroforestry, and mining activities are known to occur, details concerning these conflicts are scarce. The objective of this study is to document the human–guanaco conflict in Tacna Department, southern Peru, utilizing information from ground surveys, available remote sensing data, and the results of direct interviews. Despite the critically endangered status of the Peruvian guanaco, such information is lacking and the impact of the human–guanaco conflict has not been taken into consideration in relation to the conservation of the species.

## 2. Materials and Methods

### 2.1. Study Area

**Figure 1:**
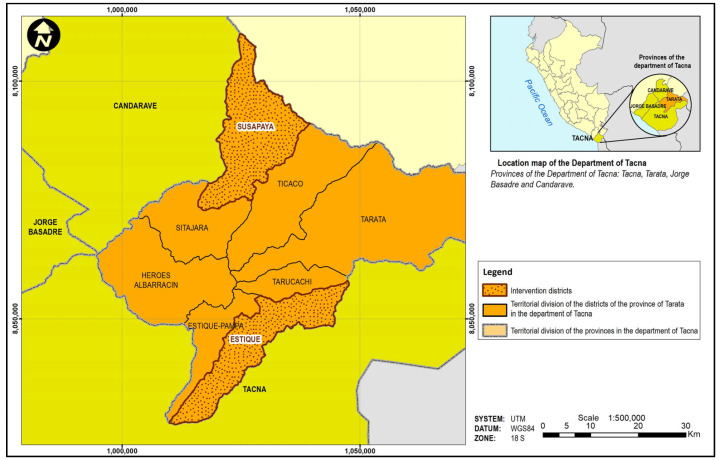
Location of the study area.

In order to document the human–guanaco conflict in extreme southern Peru, we drew on information from ground survey and remote sensing data, as well as the results of interviews of resident opinions regarding the impact of guanacos on agricultural production in the Susapaya and Estique districts of Tarata Province, Tacna department (Figure 1), located on the Sama River drainage at an average elevation of 3400 m above sea level. This extremely arid region on the Pacific slope of the Andes is classified as a Quechua or high Andean steppe ecosystem [35]. The climate is mild, with average annual temperatures between 11 and 16 °C, with maximum highs of 22–29 °C and lows of 7 to −4 °C. The scarce rainfall is concentrated during the wet season months from December to March with 54 mm on average, and less than 2 mm falling during the dry season from April to November [36]. 

**Figure 2:**
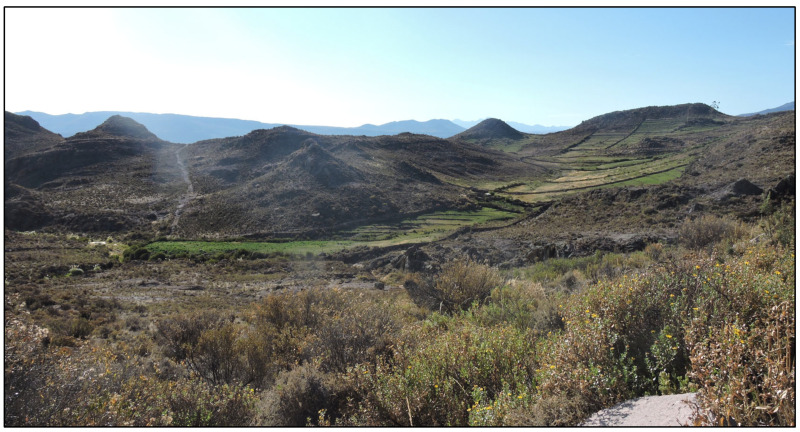
Landscape dominated by native grasses and shrubs with small agricultural plots.

The landscape is dominated by native grasses and shrubs, with small isolated agricultural plots where crops are grown for household consumption and small-scale commercialization, and alfalfa is grown to provide forage for small numbers of sheep and cattle (Figure 2). The human population in Susapaya and Estique is 518 and 240 residents, respectively [37]. Likewise, the livestock population in Estique corresponds to 57 cattle and 96 sheep, while in Susapaya 277 cattle and 268 sheep are reported [38].

### 2.2. Ground Survey

Systematic surveys were conducted to determine the presence of guanacos in the vicinity of agricultural plots in the Susapaya and Estique Districts. Data were collected from September to November 2021, towards the end of the dry season. Following the direct observation protocol developed by Castillo-Doloriert et al. [39], information on guanaco distribution was recorded and maps were prepared. Binoculars (Bushnell, China 12 X 50 mm), a monocular scope (Carl Zeiss, Wetzlar, Germany, 25–60 X 80 mm), and a drone (DJI Mavic 2, Shenzhen, China) were used in the survey. Sex determination was made by direct observation of the genitals, age assignment as young, juvenile, or adult was determined based on the relative size of individuals and their social status as a member of a family group or bachelor band, or as a solitary individual, was recorded. Survey teams consisted of two people, moving on foot or by vehicle, between 6 and 17 h seven days a week for 3 months.

### 2.3. Remote Sensing

The remote sensing data was collected during the Guanaco 2 project, financed by CONCYTEC (Grant N° 167-2017-FONDECYT) for the study of guanaco spatial ecology in Peru. Under Permit number AUT-IFS-2021-011 from SERFOR, the National Forestry, and Wildlife Service of the Ministry of Agriculture. 

GPS collars were placed on guanacos to monitor their movement. Capture protocol used a Dan-inject CO_2_ Injection rifle (Model J.M.SP.25, Kolding, Denmark) and 3 mL Dan-inject syringe darts (S300) to immobilize guanacos from a distance of 30–40 m. The agonist sedative Alfa-2-Adrenoceptor Medetomidina (Medised 20X 20 mg/mL Wildlife Pharma, Mexico City, México) and the dissociative anesthetic Ketamine (Halatal KT 10%, Montana, Lima, Peru) were used, with 0.5 mL medetomidina and 2.5 mL Ketamine in each dart. Application of the antagonist Atipamezole (Atimil 10 mg/mL Wildlife Pharma, Mexico City, México) reversed the effects of Medetomidina in approximately 40 min [40].

Selection of the guanacos that were collared was determined in large part by their tolerance of human presence, given the limited (40 m) range of the injection rifle, and strong winds in the area that diverted the darts. Advanced Telemetry System (ATS) G5 series Iridium/GPS (5D/2D) collars were used and programed to record eight fixes daily and transmit location information every 4 h. The data were received through the ATS idaq website and home range was estimated from locations accumulated over a year (almost 3000 points) using the minimum convex polygon method (MCP) [41], calculated by generating polygons where the union of all the perimeter points forms internal angles less than 180° and reducing the distribution surface of the points. Spatial distribution maps were prepared using ArcGIS 10.8 software and the ecosystem classifications and distributions from the National Ecosystem Map of Peru published by the Ministry of the Environment in 2018. The wet season was considered to last from December to March and the dry season from April to November.

### 2.4. Farmer Interviews

The survey of resident opinions concerning the impact of guanacos on agricultural production in Susapaya and Estique was conducted with the support of local elected authorities. In-person interviews were conducted with 40 farmers who had fields located near the periphery of agricultural production, close to the guanaco habitat. Information including interviewee details, agricultural activity practiced, conflict with guanacos and other wildlife, knowledge about the conservation of guanacos and confronting the problem of guanaco intrusion in agricultural plots was collected using the form in Table 1. Each interview lasted between 20 and 30 min.

We attempted to locate the owners of all the agricultural plots located close to guanaco habitat. In the Palquilla annex, Estique District, approximately ten resident families depended on produce from their fields for food. We first interviewed five farmers who were interviewed in their fields, and subsequently interviewed the five remaining at a meeting called by the local authority to discuss the guanaco problem. In the Susapaya District, we also interviewed farmers with plots located close to the guanaco habitat. In this area, alfalfa was grown to feed livestock. We initially interviewed 17 farmers in the field and, subsequently, 13 more at a communal meeting called by local authorities to discuss the guanaco conflict with SERFOR representatives. Communal meetings are a long-standing cultural tradition in the Andes where all opinions are aired, and consensus is most often reached.

## 3. Results

**Figure 3:**
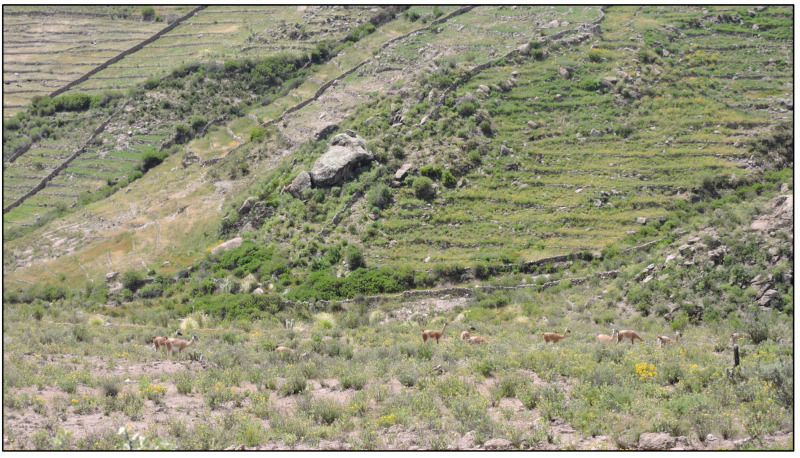
Guanaco bachelor band near agricultural plots in Susapaya. Tarata, Tacna, 2021.

**Figure 4:**
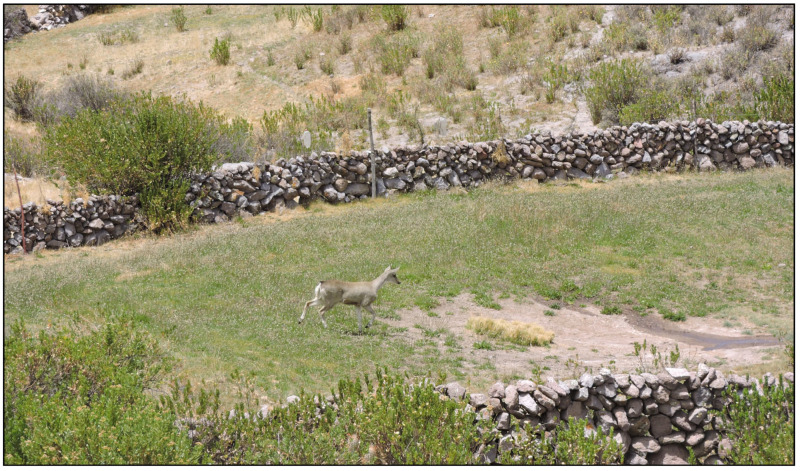
Taruka deer (*H. antisensis*) in agricultural plot in Susapaya. Tarata, Tacna, 2021.

**Figure 5:**
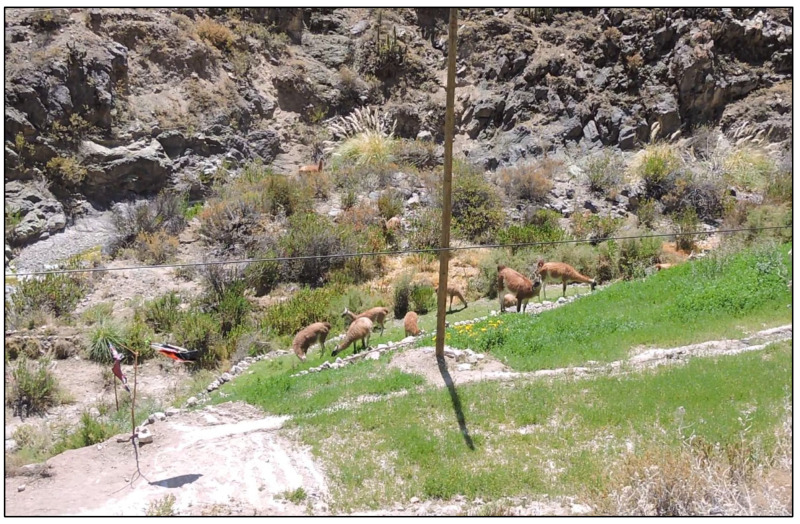
Guanaco bachelor band consuming cultivated pasture plants in Estique district.

During the ground surveys, an average of three to four guanaco sightings were recorded daily, mainly in areas of shrub steppe vegetation, occasionally very close to farmlands (Figure 3). Five sightings of guanacos in agricultural fields were recorded, including two bachelor bands and two solitary males in Susapaya, and one Bachelor band in Estique. At Susapaya, both solitary males were in close proximity to cattle. Taruka deer (*Hippocamelus antisensis*), a wild species categorized as vulnerable in Peru [42], were also observed invading crop areas in Susapaya on three occasions (Figure 4). In Estique, one bachelor band (Figure 5) was sighted twice in an isolated agricultural field.

**Figure 6:**
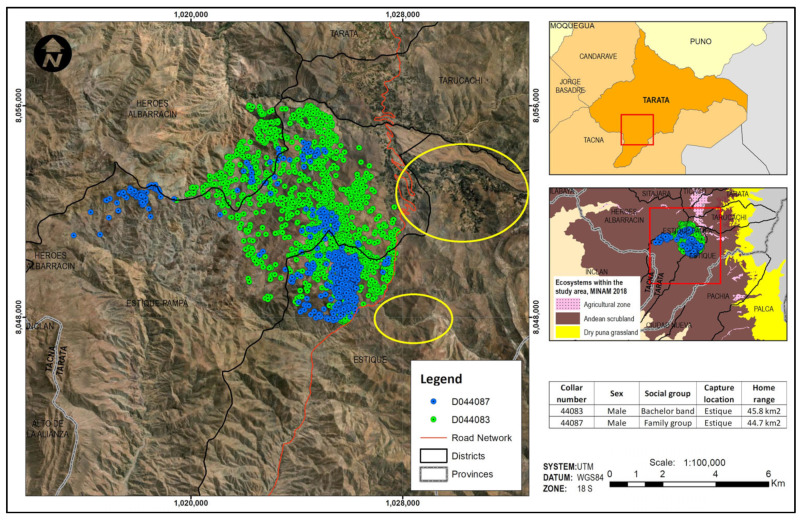
Movements of two adult male guanacos, one belonging to a troop of nonterritorial bachelor males (green) and one to a family group (blue), over a 12-month period, are located west of the Tacna–Candarave highway (orange) in Andean steppe (Andean scrubland) and away from the Estique agricultural fields located within the yellow ellipses.

**Figure 7:**
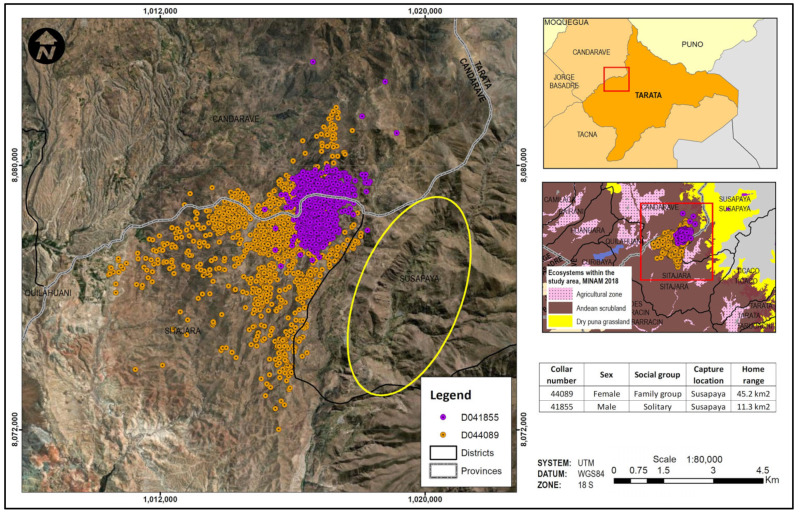
Movements of a solitary adult male (purple) and an adult female family group member (orange) over 12 months, exclusively in Andean steppe (Andean scrubland), distant from the Susapaya District agricultural fields (yellow ellipse).

The determination of guanaco movement patterns over a year was obtained through data transmitted from GPS collars. The placement of the collars was difficult due to high winds in the area and the 40 m distance limitation of the air rifle. On only 18 occasions, the combination of low wind conditions and the guanacos’ tolerance of human presence permitted attempts at immobilization. Of the eighteen, only five darts led to immobilizatio while four failed to penetrate and three failed to inject. Six were deviated before reaching the target. In total, data was obtained from four collared guanacos, two from Estique District (Figure 6) and two from Susapaya District (Figure 7). A fifth collar was placed on a guanaco in Estique District but it failed to transmit. Table 2 summarizes information about each collared animal. Although the guanacos were captured in the Andean steppe near agricultural areas, they subsequently moved deeper into the steppe where bushes provide browse for their diet. The guanacos captured in Estique moved between the Estique, Estique Pampa, and Heroes Albarracín Districts, while the guanacos captured in Susapaya moved between the Sitajara and Candarave Districts, always well away from agricultural fields (Figure 6 and Figure 7). Home range sizes were uniform, ranging from 44.7 to 45.8 km^2^, except in the case of the solitary male whose range covered only 11.3 km^2^. Guanaco home range sizes did not vary significantly from season to season, except for the female family group guanaco whose home range more than doubled during the wet season (44.4 km^2^) compared to the dry season (17.1 km^2^).

Information concerning guanaco-caused damage to crops was collected during interviews with 30 farmers from Susapaya and 10 from Estique who held agricultural plots located near guanaco territories. Fifty-five percent were men and 45% women, with an average age of 59 years (range: 31–90 years). The majority were primary (47.5%) and secondary (42.5%) school graduates, and a small number had completed post-high school technical training (5%), while the remainder (5%) had not received formal education.

Regarding agricultural activity, the average field size held by each farmer was 4 topos, with a range from 0.125 to 15 topos. A topo is a pre-Hispanic unit of measurement that is equivalent to one-third of a hectare. Their most important crop was alfalfa (70%), followed by maize (50%), potatoes (37.5%), and oregano (35%). Alfalfa is mainly used for livestock feed, the maize for both human and animal consumption, while the potatoes are for the farmers. Some alfalfa is cut to feed guinea pigs, but this crop regrows after several cuttings prior to the final harvest. Oregano is an important commercial crop in Tacna. The farmers we interviewed kept from four to twelve sheep or cattle and just over half grazed livestock in their fields, mainly cattle (57%) and sheep (22%).

In regard to conflict with wildlife, 90% of the interviewees complained that guanacos caused crop damage, while 95% responded that taruka deer were also responsible, and 66% said invasive European hares (*Lepus europaeus*) also caused damage. The worst wildlife damage was said to occur during the dry season (63% of respondents), while 32% said it occurred year-round and 5% said it happened during the wet season. Estimated crop losses ranged from 25 to 100% of production corresponding to yearly losses from USD 200 to 4000. Finally, the farmers stated that the damage to their crops had been occurring over the last 20 years, although the majority said that it had become worse during the last seven years.

Concerning knowledge about guanaco conservation, 87% of the farmers knew that guanaco hunting was prohibited, 8% were not aware of its prohibition, and 5% indicated that hunting was allowed if authorized by SERFOR. Likewise, the farmers indicated that the legal penalties for hunting guanaco were prison (50%) and fines (12.5%). However, 34% of those interviewed were unaware of these sanctions and 3.5% indicated that there was no penalty at all. Finally, 90% of those interviewed did not see any benefits or contributions coming from guanacos, although tourism (5%), use of fiber (2.5%), and meat consumption (2.5%) were mentioned as possible future benefits.

In regard to protecting their crops from guanaco and other wildlife damage, the farmers mentioned the use of fences, scarecrows, and chasing the guanacos away. Among the measures they proposed for resolving the problem were improved fencing, permanent round-the-clock watchmen, receipt of a government subsidy, use of dogs to scare guanacos away, using insecticides on the fields to kill guanacos, hunting (culling), translocating, planting special crops to attract guanacos away from agricultural fields, and finally, utilizing their fiber and meat.

Among the farmers we interviewed, 95% were aware that the Peruvian guanaco is in critical danger of extinction and knew that hunting is prohibited, but 92% did not perceive any direct or indirect benefit for either the ecosystem, or the local human population, from its conservation. Unlike the southern subspecies, very little scientific data are available for *L. g. cacsilensis*, as research on, and conservation efforts in benefit of, the Peruvian guanaco have not been prioritized.

## 4. Discussion

The documentation of the human–guanaco conflict in Tarata District, Tacna Department, southern Peru has drawn on the results of (1) a systematic ground survey to record guanaco distribution, and (2) remote sensing data from collared animals to document guanaco movement in relation to agricultural fields, as well as (3) the opinions expressed by local farmers who have lost crops to foraging guanacos.

Although satellite monitoring documented no overlap between guanaco movement and agricultural fields over 12 months, ground survey data recorded the presence of guanaco bachelor bands and solitary males in agricultural fields. Guanaco social organization is divided into territorial family groups that include an adult male, 5–7 adult females and the young of the year; roaming bachelor bands comprised of juvenile and young males who have not yet established dominance of a family group; and solitary animals, usually displaced adult family males [10,11]. The family groups occupy, and defend, the prime foraging sites, pushing non-members onto lands with poorer quality browse and eventually, with increased population growth, onto agricultural plots. Nonetheless, one of the collared guanacos from Estique belonged to a bachelor band and never entered agricultural areas for a full year, perhaps because adequate browse was available. Bachelor bands include a larger number of animals and tend to be more mobile than the territorial family groups, so may be more likely to cross into agricultural fields. In Tierra del Fuego, Chile, Moraga et al. [14] found that the average annual home range of bachelor bands was up to three times larger than those of sedentary family group members. In our study, family group and bachelor band guanacos had similar annual home range sizes, while the home range of the solitary male was four times smaller.

Guanacos are primary browsers and secondary grazers, an adaptation developed by their ancestors [43]. Analysis of guanaco movement patterns documented their preference for scrubland habitat, outside the agricultural areas. Neither of the Estique collared males crossed the Tacna–Candarave highway that separates their home ranges from the community’s agricultural fields (Figure 5), including those located in the Palquilla annex, where most guanaco damage was reported. In Estique, the only record of guanacos invading agricultural fields came from a plot on the side of the road where the guanaco home range was located.

Research carried out in populations of the *L. g. guanicoe* subspecies, southern South America, found different home range values. In Rio Negro province, Carmanchahi et al. [15] report mean home range values of 220 ± SE 51 km^2^ for six guanacos with slight size reductions during the year. Likewise, Schroeder et al. [16] report estimated seasonal guanaco home range sizes of 63–632 km^2^ for sedentary animals and 62–712 km^2^ for migratory guanacos in northern Patagonia. Our study presents data for a different subspecies in a different environment. We found home ranges of 44.7, 45.2, and 45.8 km^2^ for three adults and 11.3 km^2^ for a solitary animal in southern Peru. Clearly, home range size varies with each habitat and specific research is needed to understand the spatial ecology of the guanaco *L. g. cacsilensis*.

During the wet season, only one guanaco, the female family group member, expanded her home range 2.5 times, traveling away from areas of frequent use without crossing into agricultural fields. Her travel may reflect increased water and forage availability. Moraga et al. [14] also describe seasonal variations, where migratory guanacos expand their ranges in winter with a mean home range size 23 times larger (28.28 km^2^) than in summer (1.74 km^2^). This behavior responds to much more marked seasonal climatic changes than in the north.

Guanacos are not the only species in the region that damages crops. Destruction caused by taruka deer and European hares, especially during the dry season, was repeatedly mentioned by the farmers we interviewed. In contrast to taruka deer and guanacos, the ongoing European hare invasion is a threat to both preservation of native Andean biodiversity and economic productivity in Peru, Chile, and Argentina [44,45]. Although huemul deer were ranked above guanacos as a causal factor of crop destruction, it is possible that the recent increase in guanaco numbers makes farmers perceive guanacos to be a greater problem. This is like the situation reported by Flores et al. [15] in Patagonia, where ranchers’ negative perceptions about guanacos arise from the belief that increased wild population sizes reduce forage availability for their livestock. The resultant conflict is likely going to increase as climate change brings increasing aridity to the area [32]. Nevertheless, in the 1996 census, a total of 95 guanacos were found in the Tacna region and by 2015, 406 were reported [26,30]. In 2020, a census in the community of Susapaya found 106 guanacos concentrated in 42 km^2^ [46].

Among the actions that could help to resolve the problem, the interviewees suggested strengthened prevention measures (better fencing, permanent vigilance) supported by government subsidies, removing guanacos from the area, killing guanacos, and using guanacos for fiber and meat. These responses clearly reflect the human side of the conflict with little concern for guanaco survival. Killing guanacos, culling, is prohibited by law, and there is no conclusive evidence that such a solution will resolve or even diminish the problem in wildlife [47,48]. Although initial studies have shown normal levels of genetic variability in some Peruvian guanaco populations [6,8], it does not guarantee survival of the species. Given the very low numbers, culling or translocation could have a devastating effect on survival, and rearing guanacos in captivity for fiber and meat production cannot be an option. Unfortunately, in Peru, rearing vicuña, the other wild South American camelid “rescued from extinction”, in so-called semi-captivity “semi-cautiverio” for fiber production is an accepted practice, so it is simply assumed that such a solution should be viable for the guanaco.

## 5. Conclusions

The guanaco is the dominant native herbivore species in the Tacna region and there, as elsewhere in South America, it is considered to be a threat to agricultural productivity, although the taruka (huemul) deer and the European hare are also blamed. Guanacos are primary browsers and secondary grazers and, in the Tacna region, population growth may be a factor pushing guanaco bachelor bands beyond the Andean steppe into agricultural fields. Further research is required to document the relative impact of guanaco vs. taruka deer and hare damage to crops in Tacna. There is also an urgent need to raise awareness at the community level of the important ecosystemic role played by guanacos in order to facilitate communication and the development of mitigation measures that can reduce the conflict and produce coexistence in order to ensure the survival of the species.

## Figures and Tables

**Table 1:** Farmer interview questions.

Topic	Questions
Interviewee details	Residence
	Age of farmer
	Sex of farmer
	Education level
Agricultural activity	What size are your fields?
	What crops do you grow?
	What species of livestock do you graze in your fields?
	How many of each species of livestock do you own?
Conflict with wildlife	Which wild species damage your crops?
	What is the extent (area) of damage to your crops?
	When does the worst damage occur?
	How long have you been suffering from losses to wildlife?
	How much money do you lose annually due to crop damage caused by wildlife?
Knowledge about conservation of guanacos	Do you know if it is legal to hunt guanacos?
	Do you know what the legal penalty is for hunting guanacos?
	What benefits do guanacos contribute?
Confronting the problem	What actions have you taken to resolve the problem?
	What actions do you think might help to resolve the problem?

**Table 2:** Guanacos with collars emitting location information for twelve months, Estique and Susapaya Districts, Tacna Department. 2021–2022.

Collar Number	Sex and Age	Social Group	Capture Location *	Annual Home Range
44083	Male, adult	Bachelor band	Estique	45.8 km^2^
44087	Male, adult	Family group	Estique	44.7 km^2^
44089	Female, adult	Family group	Susapaya	45.2 km^2^
41855	Male, adult	Solitary	Susapaya	11.3 km^2^

* See Figure 6 and Figure 7 for exact locations.

## Data Availability

Data are contained within the article.
